# Supplementary material for: Identifying an oculomotor phenotype for adolescent depression with an interleaved pro- and anti-saccade task
Source: Front Psychiatry. 2026 Aug 19;17:1850946. doi: 10.3389/fpsyt.2026.1850946 (PMC13536904; doi:10.3389/fpsyt.2026.1850946)
Supplement: Supplementary file 1 [file DataSheet1.pdf]

Supplementary Material
for "Identifying an oculomotor phenotype for adolescent depression with an interleaved pro- and anti-saccade task" by Noyes *et al*.

Supplementary Methods

In the Interleaved Pro- and Anti-Saccade Task (IPAST), we measure pupil behaviour during the FIX and GAP epochs, where we see an initial constriction quickly followed by a dilation. This pattern of response (see pink curve below) occurs across all ages, and across different clinical populations[1-5]. However, the magnitude of constriction and/or dilation varies. This is because constriction and dilation responses, which are influenced by several factors (e.g., luminance, arousal, cognitive control), are driven by brain areas which develop/change with age and are impacted by neurodegenerative and psychiatric disorders[6].

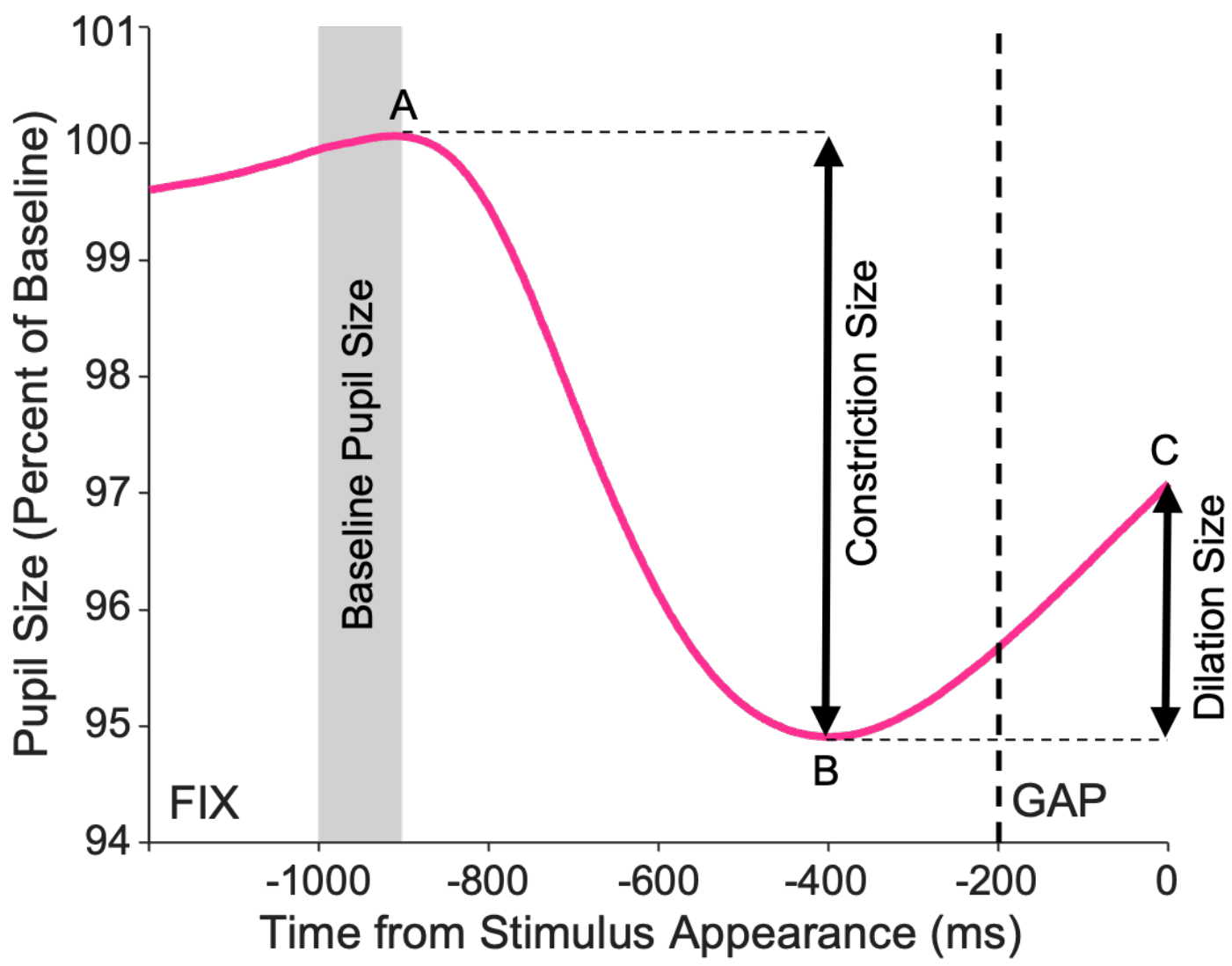

Supplementary Figure 1. Example of a typical pupillary response curve during IPAST. Baseline pupil size is calculated as the average size within the grey bar. Constriction and dilation measurements are indicated by the two arrows. Constriction is measured as the difference between B and A, and dilation is measured as the difference between C and B. Data is represented in percent change as opposed to pixels to correct for differences in baseline pupil size.

Supplementary Results - Correlations

The supplementary table below displays correlations between oculomotor behaviour and clinical scores within the depression group. Curiously, apart from the correlation between EDE-Q scores and anti-saccade reaction time, these significant correlations are in the opposite direction than expected (i.e., greater clinical impairment correlated with less oculomotor impairment). Some potential explanations behind these surprising results are suggested in the main text discussion.

Supplementary Table 1. Correlations between oculomotor behaviour and clinical scores for the depression group (n = 51).

	**BIS-30 (n = 51)**	**BSL-23 (n = 51)**	**DERS-36 (n = 51)**	**EDE-Q (n = 51)**	**GAD-7 (n = 51)**	**PHQ-9 (n = 51)**	**SBQ-R (n = 49)**
**Fixation Behaviour**							
PRO Fixation Breaks (%)	r = .010<br>p = .942	r = -.236<br>p = .096	r = -.128<br>p = .371	r = -.030<br>p = .835	r = -.431<br>**p = .002**	r = -.142<br>p = .320	r = .102<br>p = .413
ANTI Fixation Breaks (%)	r = -.069<br>p = .632	r = -.367<br>**p = .008**	r = -.132<br>p = .357	r = -.078<br>p =.586	r = -.500<br>**p < .001**	r = -.307<br>**p = .028**	r = .127<br>p = .384
Fixation Acquisition (%)	r = -.151<br>p = .291	r = .133<br>p = .353	r = .219<br>p = .123	r = .038<br>p = .793	r = .137<br>p = .339	r = .240<br>p = .090	r = -.096<br>p = .510
**Saccade Behaviour**							
PRO SRT (ms)	r = -.059<br>p = .678	r = -.117<br>p = .412	r = -.092<br>p = .520	r = .022<br>p = .880	r = .092<br>p = .522	r = -.180<br>p = .207	r = .006<br>p = .965
ANTI SRT (ms)	r = .124<br>p = .386	r = .044<br>p = .760	r = .037<br>p = .798	r = .302<br>**p = .031**	r = .097<br>p = .500	r = -.046<br>p = .751	r = .127<br>p = .386
PRO Correct Rate (%)	r = -.004<br>p = .975	r = -.066<br>p = .644	r = .048<br>p = .739	r = .003<br>p = .985	r = -.180<br>p = .207	r = .096<br>p = .503	r = -.134<br>p = .358
ANTI Correct Rate (%)	r = -.215<br>p = .130	r = -.066<br>p = .646	r = .003<br>p = .981	r = -.011<br>p = .941	r = .090<br>p = .529	r = .029<br>p = .842	r = -.102<br>p = .484
PRO Express Correct (%)	r = .150<br>p = .293	r = .160<br>p = .261	r = .163<br>p = .254	r = .047<br>p = .746	r = -.033<br>p = .819	r = .138<br>p = .334	r = .149<br>p = .308
ANTI Express Errors (%)	r = .201<br>p = .157	r = .124<br>p = .387	r = .073<br>p = .611	r = .003<br>p = .985	r = -.062<br>p = .667	r = .047<br>p = .742	r = .077<br>p = .600
ANTI Regular Errors (%)	r = .030<br>p = .833	r = -.104<br>p = .469	r = -.139<br>p = .329	r = .015<br>p = .918	r = -.054<br>p = .706	r = -.139<br>p = .332	r = .049<br>p = .737
**Pupil Behaviour**							
Baseline Pupil Size (px)	r = -.161<br>p = .259	r = .001<br>p = .997	r = -.075<br>p = .600	r = -.133<br>p = .353	r = -.028<br>p = .843	r = -.070<br>p = .624	r = -.069<br>p = .637
PRO Constriction Size (% change)	r = -.007<br>p = .960	r = .102<br>p = .477	r = .117<br>p = .414	r = -.081<br>p = .574	r = -.052<br>p = .718	r = .051<br>p = .720	r = .121<br>p = .408
PRO Dilation Size (% change)	r = -.079<br>p = .581	r = -.128<br>p = .372	r = -.168<br>p = .239	r = .008<br>p = .954	r = -.041<br>p = .776	r = -.091<br>p = .526	r = -.083<br>p = .568
ANTI Constriction Size (% change)	r = -.045<br>p = .756	r = .102<br>p = .478	r = .097<br>p = .496	r = -.101<br>p = .480	r = -.039<br>p = .785	r = .069<br>p = .633	r = .042<br>p = .773
ANTI Dilation Size (% change)	r = -.033<br>p = .820	r = -.093<br>p = .517	r = -.092<br>p = .521	r = .080<br>p = .579	r = .010<br>p = .946	r = -.019<br>p = .896	r = -.097<br>p = .506

BIS-30 = Barratt Impulsiveness Scale, BSL-23 = Borderline Symptom List, DERS-36 = Difficulties in Emotion Regulation Scale, EDE-Q = Eating Disorder Examination Questionnaire, GAD-7 = Generalized Anxiety Disorder Scale, PHQ-9 = Patient Health Questionnaire, SBQ-R = Suicide Behaviors Questionnaire Revised, PRO = pro-saccade trial, ANTI = anti-saccade trial, SRT = saccadic reaction time.

The supplementary table below displays correlations between oculomotor behaviours and clinical scores for the combined healthy control and depression groups. When adding the control group, many more significant correlations emerged. We believe this is largely driven by the large group differences in clinical scores.

Supplementary Table 2. Correlations between oculomotor behaviour and clinical scores for the entire sample (N=117).

	**BIS-30 (N = 117)**	**BSL-23 (N = 117)**	**DERS-36 (N = 117)**	**EDE-Q (N = 117)**	**GAD-7 (N = 117)**	**PHQ-9 (N = 117)**	**SBQ-R (N = 115)**
**Fixation Behaviour**							
PRO Fixation Breaks (%)	r = .219<br>**p = .018**	r = .060<br>p = .523	r = .145<br>p = .118	r = .142<br>p = .127	r = .027<br>p = .771	r = .142<br>p = .126	r = .198<br>**p = .036**
ANTI Fixation Breaks (%)	r = .187<br>**p = .044**	r = .036<br>p = .698	r = .164<br>p = .078	r = .154<br>p = .097	r = .036<br>p = .704	r = .104<br>p = .266	r = .237<br>**p = .011**
Fixation Acquisition (%)	r = -.279<br>**p = .002**	r = -.201<br>**p = .030**	r = -.178<br>p = .055	r = -.067<br>p = .475	r = -.222<br>**p = .016**	r = -.165<br>p = .075	r = -.306<br>**p < .001**
**Saccade Behaviour**							
PRO SRT (ms)	r = -.119<br>p = .200	r = -.198<br>**p = .032**	r = -.222<br>**p = .016**	r = -.105<br>p = .259	r = -.144<br>p = .123	r = -.236<br>**p = .011**	r = -.095<br>p = .311
ANTI SRT (ms)	r = .061<br>p = .511	r = .010<br>p = .915	r = .001<br>p = .993	r = .103<br>p = .270	r = .025<br>p = .788	r = -.030<br>p = .748	r = .076<br>p = .420
PRO Correct Rate (%)	r = .018<br>p = .851	r = -.071<br>p = .445	r = -.019<br>p = .836	r = -.038<br>p = .685	r = -.084<br>p = .368	r = .003<br>p = .975	r = -.109<br>p = .246
ANTI Correct Rate (%)	r = -.191<br>**p = .039**	r = -.219<br>**p = .018**	r = -.234<br>**p = .011**	r = -.076<br>p = .413	r = -.198<br>**p = .033**	r = -.189<br>**p = .041**	r = -.209<br>**p = .025**
PRO Express Correct (%)	r = .114<br>p = .122	r = .266<br>**p = .004**	r = .282<br>**p = .002**	r = .143<br>p = .124	r = .206<br>**p = .026**	r = .268<br>**p = .003**	r = .225<br>**p = .016**
ANTI Express Errors (%)	r = .159<br>p = .088	r = .248<br>**p = .007**	r = .236<br>**p = .010**	r = .100<br>p = .283	r = .200<br>**p = .031**	r = .214<br>**p = .020**	r = .197<br>**p = .035**
ANTI Regular Errors (%)	r = .094<br>p = .313	r = -.007<br>p = .941	r = .043<br>p = .643	r = -.027<br>p = .776	r = .035<br>p = .705	r = -.007<br>p = .942	r = .063<br>p = .503
**Pupil Behaviour**							
Baseline Pupil Size (px)	r = .093<br>p = .318	r = .002<br>p = .987	r = .034<br>p = .715	r = -.116<br>p = .211	r = .022<br>p = .812	r = .012<br>p = .896	r = -.026<br>p = .779
PRO Constriction Size (% change)	r = .276<br>**p = .003**	r = .196<br>p = .034	r = .248<br>**p = .007**	r = -.006<br>p = .950	r = .149<br>p = .108	r = .179<br>p = .053	r = .145<br>p = .121
PRO Dilation Size (% change)	r = -.300<br>**p = .001**	r = -.239<br>**p = .009**	r = -.295<br>**p = .001**	r = -.110<br>p = .238	r = -.249<br>**p = .007**	r = -.236<br>**p = .011**	r = -.205<br>**p = .028**
ANTI Constriction Size (% change)	r = .145<br>p = .120	r = .123<br>p = .188	r = .183<br>**p = .049**	r = -.039<br>p = .678	r = .075<br>p = .422	r = .111<br>p = .233	r = .044<br>p = .641
ANTI Dilation Size (% change)	r = -.248<br>**p = .007**	r = -.197<br>**p = .033**	r = -.222<br>**p = .016**	r = -.056<br>p = .546	r = -.187<br>**p = .044**	r = -.181<br>p = .051	r = -.186<br>**p = .046**

Supplementary Results – Comorbidity Analysis

The DEP group had a high proportion of individuals with comorbid anxiety and/or developmental disorders, which is common for this population.[7,8] We ran three separate MANOVAs for fixation (Supp. Table 3), saccade (Supp. Table 4), and pupil (Supp. Table 5) outcomes with the comorbidities (anxiety disorder, developmental disorder) as fixed factors. Comorbidity status for each class was coded as YES or NO. This analysis only identified one difference: patients with comorbid developmental disorder made less fixation breaks in pro-saccade trials than those without (6.96% vs. 12.96%; $F = 6.408$ (1, 49), $p = .015$, $\eta^2p = .120$). However, given the YES versus NO sample sizes were quite uneven, this should be investigated further with larger and more matched samples.

Supplementary Table 3. Within group-differences in fixation behaviour between patients with and without comorbidities.

	**YES**	**NO**				
	M (SD)	M (SD)	F	df	p	η²p
**Anxiety Dx (28 YES, 23 NO)**						
PRO Fixation Breaks (%)	9.46 (7.41)	7.32 (8.53)	0.44	1, 49	.836	.001
ANTI Fixation Breaks (%)	11.48 (9.27)	9.22 (12.06)	.052	1, 49	.821	.001
Fixation Acquisition (%)	74.78 (15.73)	80.87 (11.44)	1.102	1, 49	.299	.023
**Developmental Dx (38 YES, 13 NO)**						
PRO Fixation Breaks (%)	6.96 (6.15)	12.96 (10.77)	6.408	1, 49	**.015**	.120
ANTI Fixation Breaks (%)	9.34 (10.16)	13.72 (11.45)	1.769	1, 49	.190	.036
Fixation Acquisition (%)	76.95 (14.62)	79.20 (13.13)	.244	1, 49	.623	.005

YES = patients with the comorbidity, NO = patients without the comorbidity.

Supplementary Table 4. Within-group differences in saccade behaviour between patients with and without comorbidities.

	YES	NO				
	M (SD)	M (SD)	F	df	p	η^2p
**Anxiety Dx (28 YES, 23 NO)**						
PRO SRT (ms)	155.42 (31.65)	163.96 (32.09)	.005	1, 49	.942	.000
ANTI SRT (ms)	240.09 (42.01)	252.16 (40.39)	.000	1, 49	.995	.000
PRO Correct Rate (%)	97.96 (2.71)	96.99 (2.91)	.876	1, 49	.354	.018
ANTI Correct Rate (%)	71.28 (17.05)	67.19 (17.32)	.700	1, 49	.407	.015
PRO Express Correct (%)	50.74 (24.99)	46.11 (25.41)	.138	1, 49	.712	.003
ANTI Express Errors (%)	20.74 (18.62)	18.64 (14.88)	.174	1, 49	.679	.004
ANTI Regular Errors (%)	7.98 (5.20)	14.17 (11.85)	.808	1, 49	.373	.017
**Developmental Dx (38 YES, 13 NO)**						
PRO SRT (ms)	157.35 (30.94)	164.89 (34.94)	.215	1, 49	.645	.005
ANTI SRT (ms)	244.95 (42.23)	247.22 (40.17)	.008	1, 49	.930	.000
PRO Correct Rate (%)	97.86 (2.41)	96.55 (3.73)	2.102	1, 49	.154	.043
ANTI Correct Rate (%)	67.90 (18.29)	73.91 (12.70)	.866	1, 49	.357	.018
PRO Express Correct (%)	51.94 (25.48)	39.04 (21.82)	1.727	1, 49	.195	.035
ANTI Express Errors (%)	21.34 (17.38)	15.27 (15.14)	.689	1, 49	.411	.014
ANTI Regular Errors (%)	10.76 (10.29)	10.83 (5.68)	.075	1, 49	.785	.002

Supplementary Table 5. Within-group differences in pupil behaviour between patients with and without comorbidities.

	YES	NO				
	M (SD)	M (SD)	F	df	p	η^2p
**Anxiety Dx (28 YES, 23 NO)**						
Baseline Pupil Size (px)	4246.12 (1296.71)	3784.82 (1290.60)	1.043	1, 49	.312	.022
PRO Constriction Size (% change)	-4.07 (4.44)	-4.72 (6.66)	.899	1, 49	.348	.019
PRO Dilation Size (% change)	1.74 (1.81)	1.58 (2.05)	.746	1, 49	.392	.016
ANTI Constriction Size (% change)	-4.65 (5.76)	-5.52 (7.76)	1.153	1, 49	.288	.024
ANTI Dilation Size (% change)	2.05 (2.25)	2.02 (2.45)	.821	1, 49	.370	.017
**Developmental Dx (38 YES, 13 NO)**						
Baseline Pupil Size (px)	4023.65 (1328.06)	4080.26 (1272.83)	.003	1, 49	.960	.000
PRO Constriction Size (% change)	-4.26 (4.41)	-4.66 (8.12)	.212	1, 49	.647	.004
PRO Dilation Size (% change)	1.61 (1.67)	1.83 (2.56)	.548	1, 49	.463	.012
ANTI Constriction Size (% change)	-4.98 (5.58)	-5.22 (9.46)	.155	1, 49	.696	.003
ANTI Dilation Size (% change)	1.93 (1.95)	2.35 (3.24)	.774	1, 49	.383	.016

Supplementary Results - Medication Effects

Due to reported effects of psychotropic medications on oculomotor behaviour, particularly pupil dilation[9], we wanted to investigate if there were within-group differences between patients on versus off antidepressants, antipsychotics, and stimulants. While we were also interested in looking at how interactions of these medications affected oculomotor behaviour, the sample sizes for most of these interactions were too low to meaningfully investigate. The Venn diagram to the right (Supp. Fig. 2) displays the medication statuses of the participants in the depression group (n = 51).

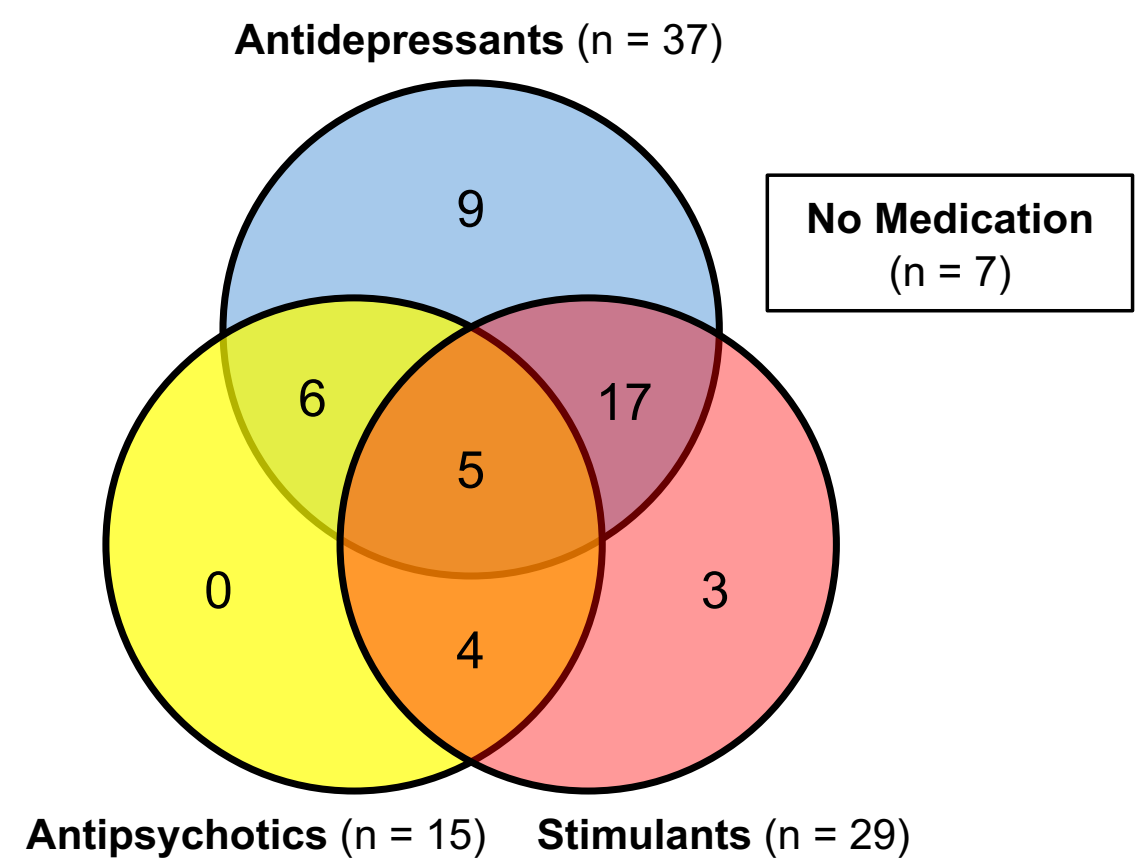

Supplementary Figure 2. Number of patients taking each class of medication.

Previous studies in our laboratory employed the ON versus OFF method when investigating effects of psychotropic medication on IPAST behaviour in youth with eating disorders[10] and a predictive saccade task in borderline personality disorder (BPD)[11]. In eating disorders, it was found that medication usage (antidepressants, antipsychotics, benzodiazepines) correlated with increased baseline pupil size but did not identify any medication effects on saccade or blink behaviour. In BPD, no effects of antidepressants, antipsychotics, or stimulants on pupil size or blink behaviour were identified.

We ran three separate MANOVAs for fixation (Supp. Table 6), saccade (Supp. Table 7), and pupil (Supp. Table 8) outcomes with the medication classes (antidepressants, antipsychotics, and stimulants) as fixed factors. Medication status for each class was coded as ON or OFF. No differences were identified for any fixation or pupil outcomes between those ON/OFF antidepressants, antipsychotics, or stimulants. Only one saccade outcome was affected by antidepressant status, with those taking antidepressants having a lower correct rate for pro-saccade trials (97.03% vs. 98.83%; $F(1,49) = 4.278$, $p = .045$, $\eta^2p = .089$). However, patients taking stimulants made significantly more correct express pro-saccades (55.4% vs. 39.8%; $F(1,49) = 11.231$, $p = .002$, $\eta^2p = .203$), as well as more express anti-saccade errors (24.1% vs. 14.1%; $F(1,49) = 8.376$, $p = .006$, $\eta^2p = .160$). These patients also had a shorter pro-saccade reaction time (152.74ms vs. 167.88ms; $F(1, 49) = 6.207$, $p = .017$, $\eta^2p = .124$), and a lower anti-saccade correct rate (65.23% vs. 74.98%; $F(1, 49) = 5.233$, $p = .027$, $\eta^2p = .106$). However, given that no other oculomotor outcomes were affected by stimulant status, we do not believe that they are driving the overall deficit in cognitive control which we see across oculomotor behaviour in the depression group.

Supplementary Table 6. Within group-differences in fixation behaviour between patients on versus off medication.

	**ON**	**OFF**				
	M (SD)	M (SD)	F	df	p	$\eta^2 p$
**Antidepressants (37 ON, 13 OFF)**						
PRO Fixation Breaks (%)	8.03 (7.74)	9.71 (8.57)	.171	1, 49	.682	.004
ANTI Fixation Breaks (%)	9.20 (7.87)	13.78 (15.54)	1.102	1, 49	.300	.024
Fixation Acquisition (%)	77.80 (14.23)	76.80 (14.47)	.166	1, 49	.686	.004
**Antipsychotics (15 ON, 35 OFF)**						
PRO Fixation Breaks (%)	7.94 (8.28)	8.72 (7.88)	.000	1, 49	.998	.000
ANTI Fixation Breaks (%)	7.95 (6.77)	11.50 (11.72)	.008	1, 49	.927	.000
Fixation Acquisition (%)	81.07 (12.77)	76.05 (14.61)	1.262	1, 49	.267	.028
**Stimulants (29 ON, 22 OFF)**						
PRO Fixation Breaks (%)	7.72 (6.21)	9.52 (9.81)	.425	1, 49	.518	.010
ANTI Fixation Breaks (%)	9.96 (7.78)	11.11 (13.57)	.586	1, 49	.448	.013
Fixation Acquisition (%)	74.24 (14.80)	81.86 (12.27)	2.69	1, 49	.108	.058

ON = patients taking the medication, OFF = patients not taking the medication.

Supplementary Table 7. Within-group differences in saccade behaviour between patients on versus off medication.

	ON	OFF				
	M (SD)	M (SD)	F	df	p	η^2p
**Antidepressants (37 ON, 13 OFF)**						
PRO SRT (ms)	161.74 (34.27)	152.74 (24.06)	.133	1, 49	.717	.003
ANTI SRT (ms)	246.37 (41.16)	243.31 (43.22)	.070	1, 49	.793	.002
PRO Correct Rate (%)	97.03 (3.08)	98.83 (1.35)	4.278	1, 49	**.045**	.089
ANTI Correct Rate (%)	70.37 (19.41)	66.96 (8.66)	.005	1, 49	.946	.000
PRO Express Correct (%)	46.72 (26.21)	53.76 (21.68)	.105	1, 49	.747	.002
ANTI Express Errors (%)	19.20 (18.38)	21.37 (12.66)	.032	1, 49	.859	.001
ANTI Regular Errors (%)	10.43 (9.72)	11.67 (8.25)	.194	1, 49	.662	.004
**Antipsychotics (15 ON, 35 OFF)**						
PRO SRT (ms)	162.19 (42.13)	158.05 (27.04)	1.656	1, 49	.205	.036
ANTI SRT (ms)	252.74 (45.92)	242.52 (39.55)	2.557	1, 49	.117	.055
PRO Correct Rate (%)	97.00 (3.70)	97.74 (2.39)	.406	1, 49	.527	.009
ANTI Correct Rate (%)	70.67 (16.60)	68.92 (17.54)	.603	1, 49	.442	.014
PRO Express Correct (%)	52.27 (27.82)	47.14 (24.04)	.710	1, 49	.404	.016
ANTI Express Errors (%)	20.37 (19.00)	19.55 (16.24)	.914	1, 49	.344	.020
ANTI Regular Errors (%)	8.96 (7.12)	11.53 (10.03)	.076	1, 49	.784	.002
**Stimulants (29 ON, 22 OFF)**						
PRO SRT (ms)	152.74 (29.37)	167.88 (33.53)	6.207	1, 49	**.017**	.124
ANTI SRT (ms)	242.73 (41.71)	249.23 (41.49)	1.714	1, 49	.197	.037
PRO Correct Rate (%)	97.87 (2.22)	97.06 (3.46)	.585	1, 49	.448	.013
ANTI Correct Rate (%)	65.23 (19.44)	74.98 (11.72)	5.233	1, 49	**.027**	.106
PRO Express Correct (%)	55.35 (25.12)	39.81 (22.51)	11.231	1, 49	**.002**	.203
ANTI Express Errors (%)	24.10 (18.57)	14.11 (12.70)	8.376	1, 49	**.006**	.160
ANTI Regular Errors (%)	10.67 (10.74)	10.91 (7.14)	.898	1, 49	.348	.020

Supplementary Table 8. Within-group differences in pupil behaviour between patients on versus off medication.

	ON	OFF				
	M (SD)	*M (SD)*	*F*	*df*	*p*	$\eta^2 p$
**Antidepressants (37 ON, 13 OFF)**						
Baseline Pupil Size (px)	4257.70 (1363.80)	3457.67 (931.15)	1.483	1, 49	.230	.033
PRO Constriction Size (% change)	-4.49 (6.08)	-4.04 (3.72)	.013	1, 49	.911	.000
PRO Dilation Size (% change)	1.89 (2.15)	1.10 (0.82)	1.221	1, 49	.275	.027
ANTI Constriction Size (% change)	-5.30 (7.50)	-4.36 (3.89)	.001	1, 49	.974	.000
ANTI Dilation Size (% change)	2.18 (2.61)	1.65 (1.25)	.238	1, 49	.628	.005
**Antipsychotics (15 ON, 35 OFF)**						
Baseline Pupil Size (px)	4385.47 (1382.47)	3893.33 (1258.22)	1.470	1, 49	.232	.032
PRO Constriction Size (% change)	-3.94 (4.36)	-4.54 (5.96)	.059	1, 49	.809	.001
PRO Dilation Size (% change)	1.16 (0.91)	1.88 (2.17)	1.439	1, 49	.237	.032
ANTI Constriction Size (% change)	-4.98 (5.07)	-5.07 (7.31)	.150	1, 49	.700	.003
ANTI Dilation Size (% change)	1.36 (0.86)	2.32 (2.67)	.758	1, 49	.389	.017
**Stimulants (29 ON, 22 OFF)**						
Baseline Pupil Size (px)	4096.75 (1226.16)	3960.74 (1420.96)	.140	1, 49	.710	.003
PRO Constriction Size (% change)	-4.29 (4.66)	-4.45 (6.56)	.439	1, 49	.511	.010
PRO Dilation Size (% change)	1.79 (1.78)	1.51 (2.09)	.265	1, 49	.609	.006
ANTI Constriction Size (% change)	-4.83 (5.91)	-5.32 (7.71)	.522	1, 49	.474	.012
ANTI Dilation Size (% change)	2.11 (2.09)	1.94 (2.64)	.001	1, 49	.976	.000

Supplementary Results – Nonparametric Testing

Given many oculomotor outcomes were not normally distributed (as expected), we re-analyzed the between-group data using a Mann-Whitney test. The results showed that all previously significant outcomes of the parametric testing (MANOVA) remained significant. All previously non-significant results remained non-significant, except for anti-saccade constriction size.

Supplementary Table 9. Between-group differences in oculomotor behaviour using non-parametric testing.

	**Z**	**p**	**Consistent with Parametric Test?**
**Fixation Behaviour**			
PRO Fixation Breaks (%)	-2.325	**.020**	Yes
ANTI Fixation Breaks (%)	-3.101	**.002**	Yes
Fixation Acquisition (%)	-3.504	**< .001**	Yes
**Saccade Behaviour**			
PRO SRT (ms)	-1.924	.054	Yes
ANTI SRT (ms)	-.176	.860	Yes
PRO Correct Rate (%)	-.147	.883	Yes
ANTI Correct Rate (%)	-2.424	**.015**	Yes
PRO Express Correct (%)	-2.328	**.020**	Yes
ANTI Express Errors (%)	-2.295	**.022**	Yes
ANTI Regular Errors (%)	-.459	.646	Yes
**Pupil Behaviour**			
Baseline Pupil Size (px)	- .434	.664	Yes
PRO Constriction Size (% change)	-2.781	**.005**	Yes
PRO Dilation Size (% change)	-3.350	**< .001**	Yes
ANTI Constriction Size (% change)	-2.177	**.030**	**No**
ANTI Dilation Size (% change)	-2.913	**.004**	Yes

Supplementary References

1. Huang J, Smorenburg ML, Yep R, et al. Age-related changes in pupil dynamics and task modulation across the healthy lifespan. *Front. Neurosci.* (2024) 18:1445727.

2. Wang CA, McInnis H, Brien DC, et al. Disruption of pupil size modulation correlates with voluntary motor preparation deficits in Parkinson's disease. *Neuropsychologia* (2016) 80:176–84.

3. Perkins JE, Janzen A, Bernhard FP, et al. Saccade, pupil, and blink responses in rapid eye movement sleep behavior disorder. *Mov. Disord.* (2021) 36:1720–6.

4. Riek HC, Visanji NP, Pitigoi IC, et al. Multimodal oculomotor assessment reveals prodromal markers of Parkinson's disease in non-manifesting LRRK2 G2019S mutation carriers. *NPJ Park. Dis.* (2024) 10:234.

5. Habibi M, Coe BC, Brien DC, et al. Saccade, pupil, and blink abnormalities in prodromal and manifest alpha-synucleinopathies. *J. Park. Dis.* (2025) 15:300-310.

6. Wang CA, Munoz DP. A circuit for pupil orienting responses: implications for cognitive modulation of pupil size. *Curr. Opin. Neurobiol.* (2015) 33:134–40.

7. Hudson, CC, Hall, L, & Harkness, KL. Prevalence of depressive disorders in individuals with autism spectrum disorder: a meta-analysis. *J. Abnorm. Child Psychol.* (2019) 47:165–175.

8. Wang, S, Stewart, TM, Ozen, I, et al. Rates of depression in children and adolescents with ADHD: a systematic review and meta-analysis. *J. Atten. Disord.* (2025) 29:924–952.

9. Constable PA, Al-Dasooqi D, Bruce R, et al. A Review of Ocular Complications Associated with Medications Used for Anxiety, Depression, and Stress. *Clin. Optom.* (2022) 14:13-25.

10. Kirkpatrick RH, Booij L, Riek HC, et al. Oculomotor behaviors in youth with an eating disorder: findings from a video-based eye tracking task. *J. Eat. Disord.* (2024) 12:121.

11. Calancie OG, Parr AC, Brien DC, et al. Motor synchronization and impulsivity in pediatric borderline personality disorder with and without attention-deficit hyperactivity disorder: an eye-tracking study of saccade, blink and pupil behavior. *Front. Neurosci.* (2023) 17:1179765.
